# Pre-Hospital Risk Factors for Inpatient Death from Severe Febrile Illness in Malian Children

**DOI:** 10.1371/journal.pone.0102530

**Published:** 2014-07-30

**Authors:** Merlin L. Willcox, Moussa I. Dicko, Bertrand Graz, Mathieu Forster, Bethany Shinkins, Chiaka Diakite, Sergio Giani, Jacques Falquet, Drissa Diallo, Eugène Dembélé

**Affiliations:** 1 Nuffield Department of Primary Care Health Sciences, University of Oxford, Oxford, Oxfordshire, United Kingdom; 2 Département de Médecine Traditionnelle, Institut National de Recherche en Santé Publique, Bamako, Mali; 3 CAM Group/Care Assessment Unit, University Institute of Social and Preventive Medicine, Lausanne University, Lausanne, Switzerland; 4 Ungava Tulattavik Health Centre, Kuujjuaq, Nunavik, Canada; 5 Aidemet ONG, Bamako, Mali; 6 Department of Plant Medicine, University of Geneva, Geneva, Switzerland; 7 Faculty of Pharmacy, Université des Sciences, Techniques et Technologies de Bamako, Bamako, Mali; 8 Department of Paediatrics, Sikasso Regional Hospital, Sikasso, Mali; Glaxo Smith Kline, Denmark

## Abstract

**Background:**

Inpatient case fatality from severe malaria remains high in much of sub-Saharan Africa. The majority of these deaths occur within 24 hours of admission, suggesting that pre-hospital management may have an impact on the risk of case fatality.

**Methods:**

Prospective cohort study, including questionnaire about pre-hospital treatment, of all 437 patients admitted with severe febrile illness (presumed to be severe malaria) to the paediatric ward in Sikasso Regional Hospital, Mali, in a two-month period.

**Findings:**

The case fatality rate was 17.4%. Coma, hypoglycaemia and respiratory distress at admission were associated with significantly higher mortality. In multiple logistic regression models and in a survival analysis to examine pre-admission risk factors for case fatality, the only consistent and significant risk factor was sex. Girls were twice as likely to die as boys (AOR 2.00, 95% CI 1.08–3.70). There was a wide variety of pre-hospital treatments used, both modern and traditional. None had a consistent impact on the risk of death across different analyses. Reported use of traditional treatments was not associated with post-admission outcome.

**Interpretation:**

Aside from well-recognised markers of severity, the main risk factor for death in this study was female sex, but this study cannot determine the reason why. Differences in pre-hospital treatments were not associated with case fatality.

## Introduction

Inpatient case fatality from severe malaria remains high in much of sub-Saharan Africa and since 2004 reports range from 2.35% to 11% [1], [2], [3], [4]. The majority of these deaths occur within 24 hours of admission, suggesting that pre-hospital management may have an impact on the risk of case fatality. Nevertheless, most studies have considered only clinical risk factors for death [1], [2], [3], [4], [5]; only a few have examined risk factors pertaining to pre-hospital management and found that delay to first treatment and use of chloroquine were associated with increased case fatality [6], [7].

In Mali, a community-based cross-sectional survey collecting retrospective data on 212 cases of presumed severe malaria in 2002 revealed that overall mortality was 17.0% (95% CI 12.2–22.7%). It did not differ significantly according to whether the patients received modern or traditional treatment [8]. Sixty-six different traditional herbal medicines were reportedly used for the treatment of malaria in this survey, of which one was selected for clinical trials and yielded promising results [9], [10], [11], [12]. In 2005 the official first-line treatment for malaria changed from chloroquine to ACTs (Artemisinin Combination Therapies: artesunate-amodiaquine and artemether-lumefantrine). The recommendation had been in force for one year at the time of this study (2006), and ACTs were supposed to be available within the public health system.

This study aimed to investigate the previous findings, and whether the change in treatment policy had made a difference. The objective was to identify pre-hospital risk factors for death from severe febrile illness (presumed severe malaria) in children.

## Methods

### Ethics statement

The study protocol was reviewed and approved by the institutional ethics committee of the Institut National de Recherche en Santé Publique, Bamako, Mali. Parents or guardians were informed about the study and were asked for their written consent, which was given for all cases included in the study.

### Study site

The study was conducted in Sikasso Hospital, in South-East Mali. Sikasso is the second largest town in Mali, and the hospital covers a population of approximately 2 million people, predominantly rural. Malaria is endemic in this area with seasonal transmission during the rainy season (July – November). One study in this area showed that 87% of febrile patients in the rainy season were positive for malaria parasites [13].

An internal audit in 2002 revealed that the inpatient case fatality rate in the paediatric ward was 24.3% (151 deaths out of 621 admissions), with 64 deaths (42.4%) attributed to malaria. Changes were then made, to have a stock of essential medications (including artemether and 10% glucose) on the ward to enable immediate treatment and to provide blood transfusions as rapidly as possible. During the present study, all inpatients with presumed severe malaria were treated according to local consensus guidelines [14]. The first-line parenteral antimalarial was intramuscular artemether, followed by artesunate/amodiaquine once the patient was able to swallow. This is because staffing levels were insufficient to permit adequate supervision of 8-hourly quinine infusions. Blood glucose was evaluated with a portable glucometer on admission and hypoglycaemia was treated. Intravenous ceftriaxone was prescribed for all comatose patients, and all patients with repeated convulsions underwent a lumbar puncture to investigate for meningitis. Staff were advised to avoid non-essential and non-evidence-based medications such as vitamins, steroids, and intravenous paracetamol. Emergency medications and medical equipment were made available on the paediatric ward so that treatment could start immediately on arrival of the patient.

Staff were motivated through participation in the development and implementation of the guidelines, regular meetings to discuss progress and difficult cases; and small bonus payments to compensate for extra work. User fees were waived for participants during the study period. The care provided to the patients in hospital was not influenced by patients' ability to pay. Therefore we could explore how differences in outcome were influenced by differences in pre-hospital care, controlling for differences in severity at the time of admission.

### Participants

All children presenting to the Paediatric Department of Sikasso Hospital with a clinical diagnosis of severe malaria (according to the admitting physician) from 28^th^ September to 30^th^ November 2006 were invited to participate. This was a prospective consecutive sample during the peak malaria season. Patients with no parasites seen on blood films were not excluded, so as not to bias the sample (because pre-admission antimalarials may make it harder to find parasites). We were careful to collect information on all patients, even those who died within a short time of admission.

### Data collection

Patients were clinically assessed, treated and stabilised as soon as possible after admission. As soon as the patient was stable, one of the interns interviewed the parent(s) or guardian of the patient using a structured questionnaire. Parents were asked about the duration of the illness before presenting to hospital; whether the patient had received any pre-hospital treatment before or after the malaria became severe, who had given this, and what the treatment was. Patients were also shown a photograph and sample of two plants commonly used for the treatment of malaria to ask whether they recognised them and whether they had used them to treat this illness. We had to rely on information given by parents because in most cases there were no written records. Patients were then followed prospectively until discharge from hospital or death.

### Statistical analysis

Data were entered using the customised data entry facility of Epi-info 6.04 (CDC, Atlanta, GA, USA) and analysed with Stata version 10.0 (Stata Corporation, College Station, TX, USA) and R statistical software (http://www.r-project.org). Descriptive statistics were computed for demographic characteristics, clinical features, pre-hospitalization malaria treatments and subsequent interventions. We examined the risk factors for death according to univariate analyses and to a logistic regression model. The model was adjusted for variables which were thought to be associated with mortality, including demographics, clinical baseline features, indicators of severe malaria and treatments. All variables were thus kept in the logistic regression, including those which were not statistically significant in univariate analyses.

A survival analysis was then carried out to test the robustness of our findings. Survival curves were estimated using the Kaplan-Meier method and differences in the curves were explored using the log-rank test. The inter-relationship of possible pre-hospital risk factors and survival was analysed using a Cox Proportional Hazards model, focusing on drug treatments received prior to hospital admission, patient demographic factors, duration of illness, and measures of the severity of the illness on arrival at the hospital. Interactions were included for weight and age, and modern and traditional treatment. Weight, age, the duration of illness and the time taken to travel to the hospital were log transformed to improve model fit. Multivariate selection of key risk factors was carried out using a stepwise procedure. For the purposes of the analytic model, patients who were discharged “cured” by the doctors were assumed to have survived for the duration of the study period. There were 12 patients who self-discharged without consent from a doctor and their survival was only assumed up to the point of discharge. In a sensitivity analysis, we verified that similar results were obtained (data not shown) when survival of those discharged “cured” was only assumed up to time of discharge. The assumption of proportional hazards over time was checked for all covariates.

## Results

There were a total of 453 admissions to the paediatric ward during the study period, of whom 437 (96%) were presumed to have severe malaria and were included in the study. None of the patients refused to participate. Their mean age was 3.5 years, 243 (55.6%) were boys, and 366 (83.8%) were aged 5 years or less. 69.5% had an interpretable blood film, of which 20% were negative for malaria parasites. 290 (66.4%) came from Sikasso town itself, while the rest came from the surrounding areas. The majority of children (287) had had at least one episode of uncomplicated malaria since the end of the last rainy season (range 0–7, median 1). 48 children had had at least one previous episode of severe malaria since the last rainy season.

The final outcomes are summarised in Table 1. The case fatality rate was 17.4%. Twelve patients were lost to follow-up or self-discharged before completion of treatment, and these were excluded from subsequent analyses, leaving a sample size of 425 patients in whom the outcome of the illness was known. Of the deaths, 34% occurred before any treatment was administered, 12% occurred less than one hour after admission, 59% occurred less than 12 hours after admission, and 86% occurred less than 24 hours after admission. There were no further deaths after 82 hours.

**Table 1:** Outcomes in the cohort of patients followed prospectively.

Outcome	Female (n)	Male (n)	Total (n)	Percent of total
Cured without sequelae	139	201	340	77.6
Deceased	45	31	76	17.4
Cured with sequelae	4	5	9	2.1
Self-discharged	6	6	12	2.7
Total	194	243	437	100

**Figure 1:**
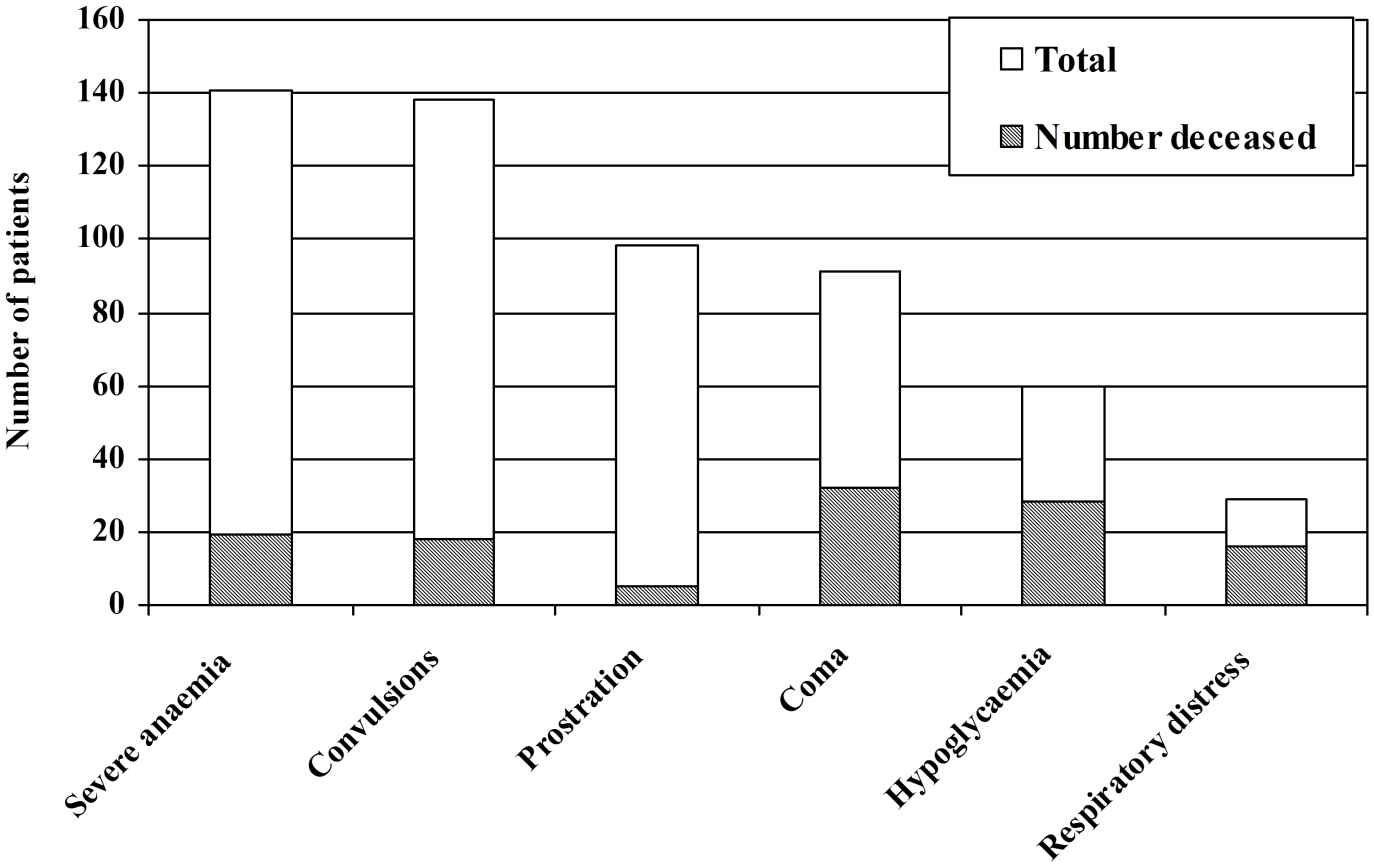
Number of patients with different features of severe malaria, and risk of death in each subgroup.

The final diagnosis at discharge remained severe malaria in 432 patients (98.9%), and 55 (12.6%) had an additional diagnosis. The most frequent comorbidity was a respiratory infection (24 cases), followed by meningitis (9) and typhoid (4). Figure 1 shows the number of patients with each subtype of severe malaria (some patients had more than one). The commonest features at admission were severe anaemia and convulsions, but case fatality in these groups was relatively low. Conversely coma, hypoglycaemia and respiratory distress at admission were more unusual but associated with significantly higher mortality (35%, 47% and 53% respectively, P<0.0001), which was confirmed in the logistic regression and survival analysis.

Of the 425 patients for whom an outcome was available, 145 (34%) had been referred to the hospital from primary health care centres in the surrounding town and region, and the rest self-presented spontaneously. Case fatality was not significantly different between those who had been referred (15.9%) compared to those who had self-presented (18.9%, p = 0.6), or between those who came from the town (19.0%) or those who came from surrounding villages (13.8%). We had information on the time taken to travel to hospital for 85 patients. For these patients, those who died had arrived in hospital significantly faster (mean of 45 minutes) compared to those who survived (mean of 90 minutes, p = 0.03). The mean reported duration of illness prior to admission was 3.7 days in patients who died and 3.9 days in patients who survived.

340 patients (82.5%) had taken some form of treatment before coming to hospital, mostly while they only had symptoms of uncomplicated malaria, before developing symptoms of severe malaria (335 patients). No pre-hospital treatment had been given to 72 patients, and in 13 cases the respondent did not know whether or not the patient had received any such treatment. Pre-hospital treatment was more often given to boys (86.5%) compared to girls (77.5%, p = 0.01), although the type of treatment did not differ significantly according to sex. In over half the cases who received a treatment, this was given by the family (table 2). Case fatality did not differ significantly according to type of provider. Only 68 patients received a treatment from a second provider, and 6 from a third provider, before coming to hospital. The commonest second provider was a health centre.

**Table 2:** Type of pre-hospital treatment(s) taken before malaria became severe, according to provider of first treatment (N  =  number of patients).

Provider of first treatment	Type of treatment:			Overall N (%)	Case fatality
	Modern only	Traditional only	Both		
Family	57	51	79	187 (45.4%)	31 (16.6%)
Health Centre	83	1	17	101 (24.5%)	16 (15.8%)
Private health professional	19	1	6	26 (6.3%)	4 (15.4%)
Traditional Healer	0	10	6	16 (3.9%)	2 (12.5%)
Street medicine vendor	1	0	3	4 (1.0%)	0 (0%)
Chinese medicine	1	0	0	1 (0.2%)	1 (100%)
None	0	0	0	77 (18.7%)	18 (23.4%)
Overall N (%)	161 (48.1%)	63 (18.8%)	111 (33.1%)	412	72 (17.5%)
Case Fatality	22 (13.7%)	15 (23.8%)	17 (15.3%)		

**Figure 2:**
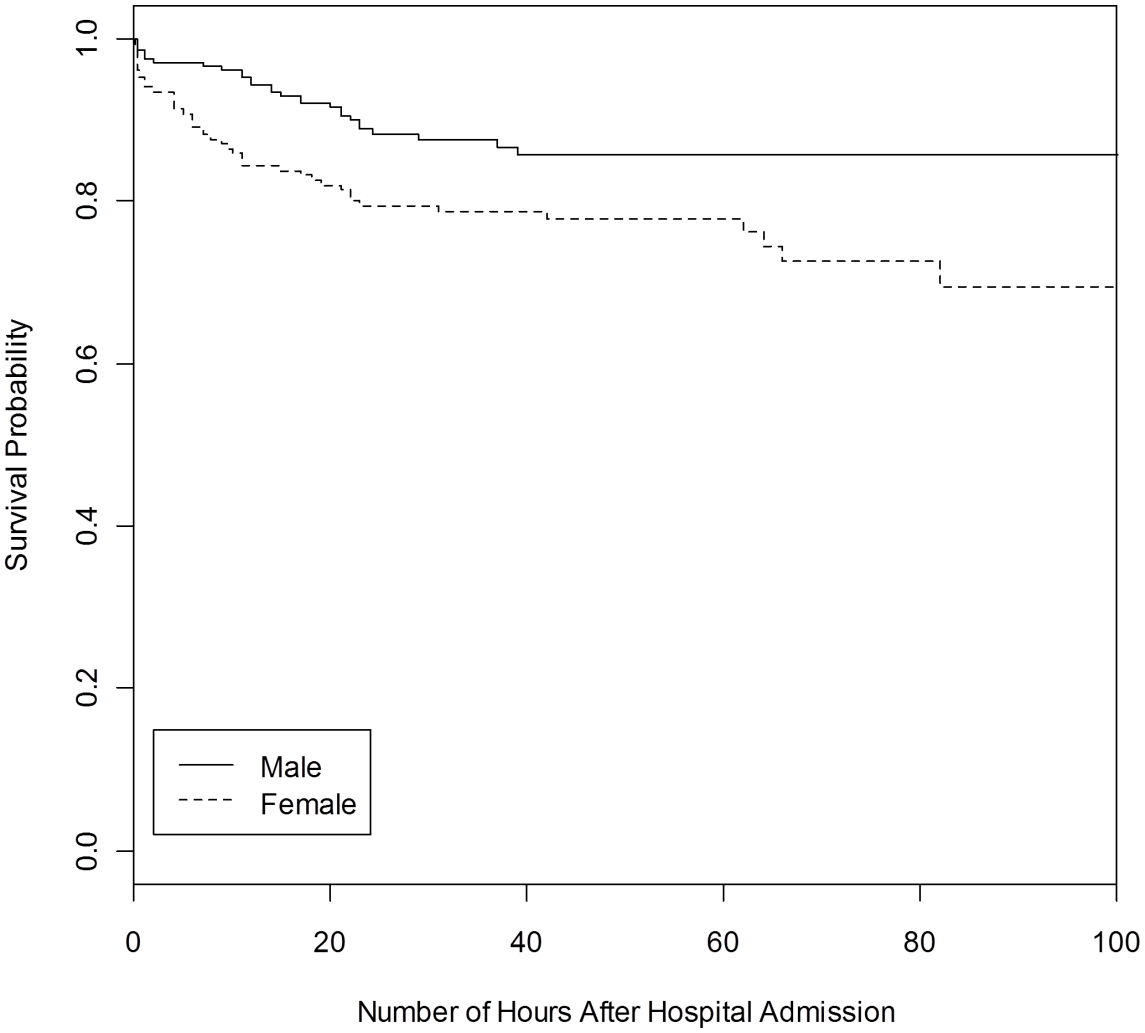
Probability of survival for 420 children with suspected malaria (males n = 233, females n = 187), truncated at 100 hours.

In multiple logistic regression models, the only consistent and significant pre-hospital risk factor for case fatality was sex. Girls were twice as likely to die as boys: of 188 girls, 45 died (23.8%), compared to 31 of 237 boys (13.1%). This difference remained significant irrespective of age, position in the family, home location (urban or rural), type of pre-hospital treatment, clinical features at admission, or duration of disease before admission (table 3). Mean reported duration of illness prior to hospitalisation was actually shorter for girls (3.5 days) than for boys (4.2 days), and girls were more likely to die within 12 hours of arrival than boys. Coma, respiratory distress and low blood glucose were all associated with significantly greater odds of case fatality. Malnutrition and positive blood films for plasmodia did not alter the results. The same results were obtained when including only those with positive blood films. Among the children who died, 69% of girls died in less than 12 hours, compared to 45% of boys (p = 0.04). Survival analysis confirmed that girls had a significantly lower chance of survival compared to boys (X*^2^ = 8.56, df = 1, p<0.01* by the log-rank test, see figure 2).

**Table 3:** Logistic Regression model of risk factors for case fatality, including socio-demographic variables, pre-hospital treatment, and clinical features at hospitalisation.

Variables	n (%)	OR (95% CI)
**Sex**		
Male	237 (13.1)	1 (ref^1^)
Female	188 (23.9)	2.00 (1.08–3.70)*
**Age**		
≤1	101 (17.8)	1 (ref)
2–3	160 (21.3)	1.00 (0.44–2.24)
≥4	164 (14.6)	0.96 (0.40–2.27)
**Sibling position**		
1	68 (14.7)	1 (ref)
2	72 (16.7)	0.67 (0.22–2.05)
3	66 (21.2)	1.83 (0.64–5.22)
≥4	216 (17.1)	1.21 (0.49–3.00)
**Town-dweller (Sikasso)**		
No	138 (13.9)	1 (ref)
Yes	284 (19.0)	1.72 (0.87–3.40)
**Previous treatment**		
None	90 (24.4)	1 (ref)
Traditional only	63 (23.8)	1.74 (0.66–4.63)
Modern only	161 (13.7)	0.88 (0.38–2.06)
Both	111 (15.3)	0.77 (0.31–1.90)
**Coma**		
No	360 (12.5)	1 (ref)
Yes	62 (45.2)	6.08 (2.96–12.49)***
**Respiratory distress**		
No	296 (11.1)	1 (ref)
Yes	126 (31.7)	3.75 (2.00–7.03)***
**Baseline glycaemia**		
≥80 g/L	360 (12.2)	1 (ref)
<80 g/L	65 (49.2)	7.22 (3.54–14.71)***
**Disease duration**		
1 day	28 (10.7)	1 (ref)
2 days	70 (17.7)	2.61 (0.49–13.99)
≥3 days	324 (17.9)	2.26 (0.48–10.65)

1 Ref  =  reference.

* *p*<0.05; ** *p*<0.01; *** *p*<0.001.

A second multiple logistic regression examined the effect of different types of pre-hospital treatment. A wide range of modern medications was taken pre-hospital, and there was an even wider range of traditional treatments (62 different herbal remedies were reported). The number of different modern treatments taken per patient was one in the majority (n = 127), two in 41 patients, three in 12 patients and four in one patient. The number of treatments taken did not affect case fatality. Apart from the broad categories of traditional only, modern only, or both, we also examined the effect of the antimalarial treatments taken by the largest numbers of people, namely chloroquine (n = 76), quinine (n = 68), amodiaquine (n = 44), co-trimoxazole (n = 31) and artemisinin derivatives or combination therapies (n = 21). In the unadjusted analysis, case fatality was reduced in those who had taken only modern treatments (OR 0.49, 95% CI 0.25–0.95), in those who had taken chloroquine in combination with traditional treatments (OR 0.17, 95% CI 0.04–0.77), and in those who had taken modern treatments other than quinine or amodiaquine. However all of these ceased to be significant in the adjusted analysis, in which the lowest risk of case fatality was in those who had taken chloroquine in combination with a traditional remedy (AOR 0.18, 95% CI 0.03–1.13), and the greatest risk of case fatality was in those who had taken amodiaquine only (AOR 2.36, 95% CI 0.67–8.29).

There was also a trend towards lower case fatality for those who had taken chloroquine only, an artemisinin derivative, or co-trimoxazole, but this did not reach statistical significance, probably because of the low absolute number of patients having taken these treatments. Treatment with amodiaquine or quinine was not associated with a reduction in case fatality.

Regarding the traditional remedies, the majority were reported only once so no analysis is possible. On direct questioning, 17 patients recognised a specimen of *Argemone mexicana* and reported its use for this illness. They had taken it for 2–4 days (mean 2.6 days) and 6 had only taken it once a day (mean 1.7 times daily). It was not associated with a reduced case fatality in this sample.

134 patients (34.5%) took some treatment after the malaria had become severe. Of these 79 had taken a modern treatment only, 23 had taken a traditional treatment only, and 32 had taken both. In these cases the traditional treatments were associated with the lowest case fatality (13.0%) compared to the modern (15.2%) or both (21.9%).

On survival analysis, chloroquine was the only drug to reveal any difference in the survival rates by the log-rank test (X*^2^ = 4.32, df = 1, p = 0.04*). However, when looking at the first 24 hours post hospital admission this drug effect is no longer evident, whereas the features of severe malaria and sex remain significant. Pre-hospital treatment with modern medicine significantly improved survival in this analysis (X*^2^ = 4.11, df = 1, p = 0.04*). Assuming the last known survival time was after the observation period (rather than time of hospital discharge) had little difference on the model. Stepwise selection of variables produced the reduced Cox Proportional Hazards model in table 4. This is broadly consistent with the findings of the regression analysis. Patients who had been pre-treated with cotrimoxazole or paracetamol were at lower risk of death, whereas those who had received amodiaquine were at higher risk of death.

**Table 4:** Variables in the Cox Proportional Hazards model after stepwise selection, assuming that all patients discharged “cured” survived until the end of the observational period.

	Multivariate proportional hazards	Lower CI	Upper CI	P-Value
log(weight in kg)	0.48	0.22	1.03	0.06
Coma	5.12	3.06	8.58	0.00
Low blood glucose (<4.4 mmol/l)	4.62	2.81	7.59	0.00
Respiratory Distress	2.76	1.67	4.55	0.00
Amodiaquine taken	2.02	0.93	4.37	0.08
Cotrimoxazole taken	0.24	0.06	1.01	0.05
Paracetamol taken	0.54	0.31	0.94	0.03
Log (Time taken to travel to hospital, in minutes)	0.54	0.40	0.74	0.00
Female sex	1.72	1.06	2.79	0.03

CI  =  confidence interval.

We also tested our assumption that patients had all received an equally good standard of care in hospital by checking whether they had been prescribed treatments according to the protocol and whether the treatment had been given. The same proportion of patients were prescribed the correct medications, and there was no barrier to receiving treatment since it was provided free of charge on the ward. 10 girls and 6 boys died before receiving any treatment (reflecting the more rapid rate of death in girls). Ten patients (of 122) who had been prescribed a blood transfusion did not receive one, and eight of these were girls (odds ratio for a girl receiving a transfusion compared to a boy  =  0.17, 95% CI 0.02–0.91). Numbers were insufficient for inclusion in the model. Of the 8 girls who did not receive a transfusion, 5 died, one was lost to follow-up and two survived. Of the two boys, one died and one survived. Of the 10 children who did not receive transfusions, 6 died. Looking just at the children who died, 17 had been prescribed a transfusion, which was received by 11 (4 of 5 boys and 7 of 12 girls). This does explain part but not all of the sex difference in death rates. Even excluding these deaths, there is a significant excess of female deaths. Sensitivity analyses demonstrated that the difference between boys and girls remains even with the lack-of-transfusion deaths removed.

## Discussion

### Principle findings

As 86% of deaths occurred within 24 hours of admission, pre-hospital treatment is likely to have the biggest impact on reducing case fatality. The commonest pre-hospital treatments were traditional (n = 174), paracetamol (144), chloroquine (76) and quinine (68). These were all widely available at the time of the study. However none of the pre-hospital treatments had an effect which reached statistical significance in the adjusted logistic regression model. It is possible that some of these effects would become statistically significant with a larger sample size, as the number of patients having taken any particular treatment was relatively small. Furthermore no information is available about dose, duration, compliance, sequence and quality of the treatments. Traditional treatments did not significantly alter the effect of modern treatments (OR 0.88 “modern only” versus 0.77 “both treatments” as compared to “no treatment”). The extreme diversity of different treatments and combinations (modern-modern, traditional-modern) shows that official treatment recommendations were not followed most of the time. Although chloroquine is no longer recommended as a treatment for malaria in Mali, it seemed to be more protective than amodiaquine, which has been increasingly used as monotherapy when ACTs are out of stock. This may have led to an increase in amodiaquine resistance which may explain why this treatment alone seemed the least effective. It is interesting that co-trimoxazole seemed protective, as it has both antimalarial and antibiotic properties.

The major factor associated with survival was sex: the case fatality rate in girls was twice that in boys, even after adjusting for all the other risk factors we could think of. Data on duration of illness prior to hospitalisation and time to death imply that girls deteriorated faster than boys. Retrospective data from hospital records did not reveal the same discrepancy between the sexes. Hospital policy was to exclude from their statistics those patients who died before receiving treatment. In our cohort we were particularly careful to include all children who arrived in the paediatric department with severe febrile illness (even if they died very soon after arrival), and of these, twice as many girls as boys were excluded from the official statistics. Other studies which have not attempted to include all patients may therefore have missed a difference in mortality between the sexes [15]. Demographic and Health Survey data on infant mortality in Mali found that male infants were at greater risk of death from malaria than female infants [16]; but this trend may be reversed for older children. Sociological factors are the most obvious explanation for the difference in survival, for example girls might be less well nourished, and have less resources spent on them (for treatment) than boys in a male-dominated society. Indeed the fact that this cohort included significantly more boys than girls (55.6% vs 44.4%) suggests that families are more likely to bring boys to hospital than girls, because there is no reason to believe that incidence of severe malaria is greater in boys than in girls. Girls may only be brought to hospital when they are more seriously ill than is the case for boys. The only significant differences we could find between management of boys and girls in our dataset was that boys were slightly more likely to have received a pre-hospital treatment (although the type of treatment did not differ), and more likely to receive a transfusion when it was prescribed. These are important findings and should be investigated further, but they do not fully explain the large observed difference in case fatality.

There was no significant association in any of the models between case fatality and reported pre-hospital duration of illness, or treatment provider. Traditional treatments did not significantly worsen prognosis, and may indeed have improved prognosis when taken together with chloroquine. It would be interesting to research this further, as there are indications that some herbal medicines can reverse resistance to chloroquine [17]. These findings differ from those of other studies; in Sudan it was found that delay in seeking treatment was a risk factor for mortality [7]. A study in Nigeria found that chloroquine was associated with a worse prognosis [6]. Some studies claim that that patients consulting traditional healers or taking traditional medicines have a worse outcome [18] but this is not borne out by our findings. Since most patients (64%) presented at the hospital after a disease duration of three days or more, it was not statistically possible to assess the possible protective effect of an early presentation. The odds ratio for death in children presenting three or more days after the start of illness had a wide confidence interval (OR 2.26, 95% CI 0.48–10.65). Furthermore living in Sikasso town (relatively close to the hospital) and a short time of travel to the hospital were associated with a worse prognosis, implying that patients with rapidly progressive illness from further afield die before arriving at the hospital.

Mortality was greatest for patients with respiratory distress, hypoglycaemia or coma. The effect of hypoglycaemia is discussed in more detail in a separate paper [14]. These findings agree with the results of other studies [5], [19], [20]. We investigated the treatment of hypoglycaemia with sublingual sugar in a separate study [21], but did not implement the findings in this study (infusions of intravenous glucose were used as per the consensus guidelines). It is possible that pre-hospital use of sublingual sugar on a routine basis could further reduce mortality in patients with hypoglycaemia.

### Strengths and weaknesses of the study

This is one of the first studies to examine the impact of pre-hospital treatment on case fatality for children admitted with severe malaria. We made every effort to implement a best-practice standard protocol for inpatient management of severe malaria, and treatment was given free of charge. This may otherwise have been an important confounding factor as poorer patients may not have been able to afford the full treatment. Comparison with hospital statistics from previous years suggests that this intervention in itself reduced case fatality below the previous level (although the inclusion criteria were not directly comparable). Another study has shown that an intervention to improve quality of care in the hospital, and to make care free of charge, can reduce inpatient case fatality [22]. However we were unable to completely standardise blood transfusion, because no patient can receive a transfusion until a relative or friend has donated blood and our data suggests that it was harder to find blood donors for girls than for boys.

The fact that we recruited only hospitalised patients means that the sample is biased, because the majority of patients with severe malaria do not present to hospital [8]. However we believe that more patients presented than usual, because fees for treatment were waived. The number of malaria admissions was greater than in the previous malaria season. All those included represent a failure of pre-hospital treatment, which should aim to prevent not only death but also severe malaria. Therefore community studies would be better for estimating the effectiveness of treatments in the whole population. However such studies would have to be extremely large to gather information on the relatively small proportion of deaths. Even in this study of 425 patients, the number in each treatment sub-category was small because of the high diversity of treatments used, so there was insufficient power to detect differences between individual treatments with confidence and statistical significance.

The diagnosis was left to the admitting physician, without defining precise criteria for “severe malaria”. Almost every severe febrile illness was labelled as “severe malaria”, and the diagnosis at discharge was still “severe malaria” in all but 4 patients. A study in the community at the same time found that 87% of patients presenting with symptoms of fever had a blood film positive for malaria [13]. Although the population is not strictly comparable, the rate of over-diagnosis was probably <15%.

There is a risk that answers to the questionnaire may not have been accurate, because the patient's relatives may have feared criticism, or because they were not fully aware of the treatment taken, or simply because their memory was imperfect. The history was taken at admission, although for some cases it was not possible to take the history before the patient died (as 34% died less than one hour after arrival). In 16.2% of the cases, the respondent was not one of the parents of the child. The fact that the study took place in a hospital, and the questionnaire was administered by an intern, may have influenced the replies. We relied on the memory and honesty of parents and did not verify the drug history through urine testing, so we have no information on compliance or quality of the drugs taken. It is possible that some of them may have been of poor quality, out of date, or counterfeit. As this is an observational study, “confounding by indication” cannot be excluded, even while controlling for many clinical factors. It is also impossible to accurately standardise the time from symptom onset to arrival at hospital.

### Meaning of the study

These findings have several implications for public health policies. In spite of the improvements we made to hospital care, the majority of deaths occurred within a short time of admission. It seems that, like meningitis in European settings, the infection can progress very rapidly from a seemingly innocuous prodrome, and can unexpectedly become severe. We did not ask care-givers how long after the start of the illness they gave the first treatment, and this would be interesting to include in future studies. However it seems logical that the earlier an effective treatment is given, the better the outcome should be.

As the first source of treatment was the family in over 55% of cases, parents and families need training on when and how to administer “home-based management of malaria”. This could be delivered alongside interventions to prevent childhood diseases, such as vaccination and distribution of insecticide-treated bednets. Efficacy alone is not sufficient to prevent death; effective treatments must be widely available, for girls as well as for boys.

### Unanswered questions and future research

Further research is needed to show whether a concerted and integrated intervention in home-based prevention and management of childhood febrile illness can indeed reduce case fatality, and how all treatment providers can collaborate to make this a reality. There is a lot of scope for further research on traditional medicines, and for their improvement. As hypoglycaemia is also an important risk factor for death, early administration of sugar may also be important [14]. Further studies are needed to investigate compliance with different treatments, determinants of choice, and treatment sequence. It would also be useful to collect information on severity of symptoms before hospitalisation.

Further research is needed to clarify why the case fatality was twice as high in girls as in boys, whether this finding is more widely generalisable to other settings, and what interventions could be envisaged to address this inequality. Higher case fatality in girls with severe malaria has also been noticed in a similar study in Gabon [1] and in Ghana it has been reported that girls are more likely to be treated at home, whereas boys are more likely to be taken to a health facility [23].

Although improvements in quality of care at the hospital can reduce inpatient case fatality, the real challenge is to investigate how these improvements can be sustained beyond the period of the research study, and how capacity can be increased to meet the increased demand for hospital treatment. This will probably require continued motivation of staff through regular audit or “significant event analysis”, and possibly a degree of external supervision, as well as additional financial and human resources.

### Conclusion

Aside from well-recognised markers of severity, the main risk factor for death in this study was female sex, which could be a proxy for other socio-cultural determinants. Differences in pre-hospital treatments were not associated with case fatality in this study.
